# Supplementary material for: Diffuse white matter pathology in multiple sclerosis during treatment with dimethyl fumarate—An observational study of changes in normal-appearing white matter using proton magnetic resonance spectroscopy
Source: PLoS One. 2024 Oct 21;19(10):e0309547. doi: 10.1371/journal.pone.0309547 (PMC11493296; doi:10.1371/journal.pone.0309547)
Supplement: S1 Table — (DOCX) [file pone.0309547.s001.docx]

**Supplemental table 1. MRI clinical protocol**

| Protocol name | T1w_IR_SE | FLAIR | T2w | Gd T1w_IR |
| --- | --- | --- | --- | --- |
| Weighting | T1w | FLAIR | T2w | T1w |
| Image type | 2D Spin Echo | 2D Spin Echo | 2D Spin Echo | 2D Spin Echo |
| Acquisition time | 06:40 | 06:36 | 03:36 | 06:40 |
| Orientation | Tra | Tra | Tra | Tra |
| Alignment | ACPC | ACPC | ACPC | ACPC |
| Voxel size | 0.575*0.72*3 | 0.7*0.98*3 | 0.6*0.6*3 | 0.575*0.72*3 |
| Repetition time | 2000 | 9000 | 4000 | 2000 |
| Echo time | 20 | 120 | 109 | 20 |
| Inversion time | 800 | 2500 | - | 800 |
| Flip Angle | 90 | 90/180 | 90/180 | 90 |
| Number of excitations averaged | 1 | 1 | 1 | 1 |
| Field of view | 230*183*142 | 230*183*142 | 230*230*142 | 230*183*142 |
| Matrix size | 400*251*43 | 328*187*43 | 384*384*43 | 400*251*43 |
| Parallel imaging | 1.5 SENSE | 1.5 SENSE | No | 1.5 SENSE |
| Contrast enhancement | NO | NO | NO | Yes |
