# Supplementary material for: Diffuse white matter pathology in multiple sclerosis during treatment with dimethyl fumarate—An observational study of changes in normal-appearing white matter using proton magnetic resonance spectroscopy
Source: PLoS One. 2024 Oct 21;19(10):e0309547. doi: 10.1371/journal.pone.0309547 (PMC11493296; doi:10.1371/journal.pone.0309547)
Supplement: S2 Table — (DOCX) [file pone.0309547.s002.docx]

| **Supplemental table 2. MRS patient cohort at each time point and reason for missing MRS data.** | | | | |
| --- | --- | --- | --- | --- |
| Pat id | Baseline | 1 year | 3 years | Reason for missing MRS data |
| 1 |  | x | x | MRS data acquisition failure at baseline |
| 2 |  | x | x | MRS data acquisition failure at baseline |
| 3 | x | x | x |  |
| 4 |  |  | x | MRS data acquisition failure at baseline and one year |
| 5 | x | x |  | MRS data acquisition failure at three years |
| 6 | x | x | x |  |
| 7 | x | x | x |  |
| 8 | x | x |  | Treatment switch due to MRI activity |
| 9 |  |  | x | MRS data acquisition failure at baseline and one year |
| 10 |  | x | x | MRS data aquisition failure at baseline |
| 11 | x | x | x |  |
| 12 | x | x | x |  |
| 13 | x | x |  | Treatment switch due to side effects of DMF treatment |
| 14 | x | x | x |  |
| 15 | x | x |  | Treatment switch due to MRI activity |
| 16 | x | x | x |  |
| 17 | x | x |  | Treatment switch due to MRI activity |
| 18 | x | x |  | Treatment switch due to MRI activity |
| 19 |  |  | x | MRS data acquisition failure at baseline and one year |
| 20 | x | x |  | Treatment switch due to MRI activity |
| 21 | x | x | x |  |
| 22 |  |  | x | MRS data acquisition failure at baseline and one year |
| 23 | x | x |  | DMF treatment stop because of pregnancy |
| 24 | x | x | x |  |
| 25 | x | x | x |  |
| 26 | x | x | x |  |
