# Supplementary material for: Diffuse white matter pathology in multiple sclerosis during treatment with dimethyl fumarate—An observational study of changes in normal-appearing white matter using proton magnetic resonance spectroscopy
Source: PLoS One. 2024 Oct 21;19(10):e0309547. doi: 10.1371/journal.pone.0309547 (PMC11493296; doi:10.1371/journal.pone.0309547)
Supplement: S3 Table — (DOCX) [file pone.0309547.s003.docx]

| **Supplemental table 3. Metabolite concentrations and ratios in relation to disease activity during follow-up.** | | | | | | | | | | |
| --- | --- | --- | --- | --- | --- | --- | --- | --- | --- | --- |
|  | At 1-year follow-up | | | | | At 3-years follow-up | | | | |
|  |  |  |  |  |  |  |  |  |  |  |
| Metabolites | NEDA (0-1 year) | | EDA (0-1 year) | |  | NEDA (0-3 years) | | EDA (0-3 years) | |  |
|  | n=11 | | n=11 | |  | n=8 | | n=10 | |  |
|  | mean | SD | mean | SD | p† | mean | SD | mean | SD | p† |
| tNA | 11.60 | 1.15 | 11.06 | 0.64 | 0.19 | 11.05 | 1.16 | 11.02 | 1.32 | 0.97 |
| *m*Ins | 5.64 | 0.99 | 5.96 | 1.01 | 0.46 | 6.00 | 1.99 | 5.90 | 2.35 | 0.92 |
| tCho | 2.25 | 0.25 | 2.28 | 0.32 | 0.81 | 2.29 | 0.17 | 2.33 | 0.32 | 0.72 |
| tCr | 6.01 | 0.53 | 6.14 | 0.39 | 0.54 | 6.29 | 0.42 | 6.47 | 0.54 | 0.46 |
| Glx | 10.89 | 1.42 | 10.47 | 1.58 | 0.52 | 10.09 | 1.92 | 10.96 | 2.42 | 0.42 |
| Lac | 0.71 | 0.34 | 1.01 | 0.74 | 0.24 | 1.12 | 0.72 | 1.45 | 0.92 | 0.42 |
|  |  |  |  |  |  |  |  |  |  |  |
| **Ratios** |  |  |  |  |  |  |  |  |  |  |
| tNA/tCr | 1.94 | 0.20 | 1.81 | 0.18 | 0.15 | 1.77 | 0.23 | 1.71 | 0.24 | 0.66 |
| *m*Ins/tNA | 0.50 | 0.13 | 0.54 | 0.10 | 0.36 | 0.56 | 0.23 | 0.56 | 0.26 | 0.99 |
| tCho/tNA | 0.19 | 0.02 | 0.21 | 0.03 | 0.34 | 0.21 | 0.03 | 0.21 | 0.03 | 0.81 |
| Glx/tNA | 0.95 | 0.18 | 0.95 | 0.13 | 0.94 | 0.93 | 0.25 | 1.02 | 0.33 | 0.54 |

† p refers to independent samples t-test comparing metabolite concentrations and ratios between patients with no evidence of disease

activity (NEDA) and patients with evidence of disease activity (EDA) during the one- and three-years follow-up, respectively.

tNA, total N-acetylaspartate and N-acetylaspartylglutamate; *m*Ins, *myo*-inositol; tCho, total choline; tCr, total creatine; Glx, the sum of glutamate and glutamine; Lac, lactate
