# Supplementary material for: Diffuse white matter pathology in multiple sclerosis during treatment with dimethyl fumarate—An observational study of changes in normal-appearing white matter using proton magnetic resonance spectroscopy
Source: PLoS One. 2024 Oct 21;19(10):e0309547. doi: 10.1371/journal.pone.0309547 (PMC11493296; doi:10.1371/journal.pone.0309547)
Supplement: Attachment — Submitted filename: Response to Reviewers.docx [file pone.0309547.s005.docx]

**Response to the Editor and Reviewers**

1. Please ensure that your manuscript meets PLOS ONE's style requirements, including those for file naming. The PLOS ONE style templates can be found at 
<https://journals.plos.org/plosone/s/file?id=wjVg/PLOSOne_formatting_sample_main_body.pdf> and 
<https://journals.plos.org/plosone/s/file?id=ba62/PLOSOne_formatting_sample_title_authors_affiliations.pdf>

Author response: This has been done.

2. PLOS requires an ORCID iD for the corresponding author in Editorial Manager on papers submitted after December 6th, 2016. Please ensure that you have an ORCID iD and that it is validated in Editorial Manager. To do this, go to ‘Update my Information’ (in the upper left-hand corner of the main menu), and click on the Fetch/Validate link next to the ORCID field. This will take you to the ORCID site and allow you to create a new iD or authenticate a pre-existing iD in Editorial Manager. Please see the following video for instructions on linking an ORCID iD to your Editorial Manager account: <https://www.youtube.com/watch?v=_xcclfuvtxQ>

Author response: The ORCID ID of the corresponding author has been added.

3. Note from Emily Chenette, Editor in Chief of PLOS ONE, and Iain Hrynaszkiewicz, Director of Open Research Solutions at PLOS: Did you know that depositing data in a repository is associated with up to a 25% citation advantage (<https://doi.org/10.1371/journal.pone.0230416>)? If you’ve not already done so, consider depositing your raw data in a repository to ensure your work is read, appreciated and cited by the largest possible audience. You’ll also earn an Accessible Data icon on your published paper if you deposit your data in any participating repository (<https://plos.org/open-science/open-data/#accessible-data>).

Author response: The raw data for this study will be uploaded and available as a Supplemental file to the manuscript (S4 Raw data).

4. We note that the grant information you provided in the ‘Funding Information’ and ‘Financial Disclosure’ sections do not match.

When you resubmit, please ensure that you provide the correct grant numbers for the awards you received for your study in the ‘Funding Information’ section.

Author response: We have updated the information in the Funding section to:

“The study was funded by the Medical Research Council of Southeast Sweden, the Swedish Foundation for MS Research, ALF grants and Region Östergötland. The funding sources were not involved in the collection, analysis, and interpretation of data or in the writing of the report and the decision to submit the article for publication.”

5. In the online submission form, you indicated that the data underlying the results presented in the study are available from the corresponding author upon request (Johan Mellergård).
All PLOS journals now require all data underlying the findings described in their manuscript to be freely available to other researchers, either 1. In a public repository, 2. Within the manuscript itself, or 3. Uploaded as supplementary information.
This policy applies to all data except where public deposition would breach compliance with the protocol approved by your research ethics board. If your data cannot be made publicly available for ethical or legal reasons (e.g., public availability would compromise patient privacy), please explain your reasons on resubmission and your exemption request will be escalated for approval.

Author response: As mentioned above the raw data for this study will be uploaded as supplementary information.

6. We note that you have included the phrase “data not shown” in your manuscript. Unfortunately, this does not meet our data sharing requirements. PLOS does not permit references to inaccessible data. We require that authors provide all relevant data within the paper, Supporting Information files, or in an acceptable, public repository. Please add a citation to support this phrase or upload the data that corresponds with these findings to a stable repository (such as Figshare or Dryad) and provide and URLs, DOIs, or accession numbers that may be used to access these data. Or, if the data are not a core part of the research being presented in your study, we ask that you remove the phrase that refers to these data.

Author response: We have removed the phrase “data not shown” and inform about the Supplemental information available.

7. Please include captions for your Supporting Information files at the end of your manuscript, and update any in-text citations to match accordingly. Please see our Supporting Information guidelines for more information: <http://journals.plos.org/plosone/s/supporting-information>.

Additional Editor Comments:

My specific comment to the authors is a statistical point. The authors compared the data of MRS between baseline and 1 year by paired t-test. The number of the subjects were decreased in the follow-up study because of drop-out. In such case the data should be compared between groups (not paired!) using t-test (n = 26 vs n =19). The data analysis between the baseline and 3 year follow-up should be compared by t-test (n = 26 vs n = 11).

Author response: Because of the dropouts we chose to use paired comparisons with t-tests since we claim that if comparison between groups is performed it would introduce a selection bias in the analysis. This is because the two groups are not comparable as the dropouts were partly due to a deficient effect of dimethyl fumarate with disease breakthroughs as for example the occurrence of new MRI lesions (see Supplemental table S2 for details). A statistical requirement for comparing two groups is that they are unrelated to each other, but in our case there is a major overlap with the majority of studypersons included in both the baseline and follow-up group. We therefore mean that paired comparisons including only study persons with measurements at both time points are more adequate to evaluate the effect of dimethyl fumarate in this context.

5. Review Comments to the Author

Reviewer #1: The manuscript by Tisell et al investigates the effect of dimethyl fumarate (DMF) in MS patients longitudinally.
MRS and MRI data were acquired at baseline (before DMF), 1 and 3 years follow up after treatment with DMF.
Results show that mIns was higher at baseline in MS vs controls subjects. No change in metabolites concentration were observed in the follow up scans.

The study looks promising, however, many important information on the MR spectroscopy section are missing. I would suggest the authors to follow the MRS concensus guidelines (<https://analyticalsciencejournals.onlinelibrary.wiley.com/toc/10991492/2021/34/5>) in order to allow data reproducibility.

1) Can the author representative MR spectra at each time points between one patient and control to visualize the data quality and see that metabolite changes? The placement of the VOI in one subject will also help.

Author response: A figure showing a typical spectrum for each time point and VOI placement has been added to the manuscript (Figure 1) in the methods section.

2) How B0 shim on the VOI carried out? If yes, please mention and the mean linewidth between cohorts. Did the authors use an criteria to reject bad spectra, e.g. if linewidth is very broad or artifact in data?
Any post-processing carried out?

Author response: The scanner PB-auto method with first order shims was used for B0 shimming. Mean linewidth calculated using LCModel was 0.042 ppm. All spectra were visually inspected but no fixed criteria for rejecting spectra was set. Maximum FWHM was 0.057 ppm.

3) A recent paper reported that DMF promotes glycolysis and increase glycine (Gly) <https://www.nature.com/articles/s42003-023-05443-4>]. Did the basis set include Gly basis spectrum? What other metabolites were included in the basis set? Was the macromolecule measured or simulated?

Author response: We did not include glycine in the basis set, since at the time of the study this was not included in the standard basis set. The basis set we used was provided by Stephen Provencher and included 18 basis metabolites. L-Alanine, Aspartate, Creatine, Phosphocreatine, Gamma-Aminobutyric Acid (GABA), Glucose, Glutamine, Glutamate, Glutathione, Glycerophocholine, Phosphocholine, *myo*-Inositol, L-Lactate, N-Acetylaspartate, N-Acetylaspartylglutamate, *scyllo*-Inositol, Taurine and Guanidinoacetate. These metabolites were simulated. Moreover, simulated lipid and macromolecules were also included and a correction term for CrCH2 singlet.

4) Can the authors elaborate more why they choose the control group as patients with CNS inflammatory disease instead of healthy controls?

Author response: It is true that the control group we used had symptoms suggestive of CNS inflammatory disease, however, after the work-up with MRI and CSF analyses, we could *not* find signs of inflammatory CNS disease in these patients. The symptoms they presented were therefore considered unspecific. In a clinical context we think it is of more interest to establish metabolite patterns that can differentiate an inflammatory CNS disease like MS from unspecific findings in patients seeking medical care with different neurologic symptoms. Such metabolite patterns would have a greater potential to reflect specific mechanisms of MS than patterns differentiating MS patients from healthy controls. We have added these sentences in the paragraph in the methods section:

“This type of control group was used because the study was done in a clinical context where we aimed to find metabolite patterns in NAWM that could differentiate MS inflammation from unspecific findings in patients seeking medical care with different neurologic symptoms. Such metabolite patterns would have a greater potential to reflect specific inflammatory mechanisms of MS and would be of more value in a clinical context, than patterns differentiating MS patients from healthy controls.”

5) Does lactate really increase or is LCmodel fitting lipid signal as lactate? It is hard to tell due to missing MRS data.

Author response: It is correct that separating lipids and lactate using short echo time MRS is difficult. However, we believe that changes in normal appearing white matter are more likely to be due to changes in lactate levels than in free lipid levels that we normally only see in active lesions or malignant tumors.

6) in introduction MRS is referred to as "advanced MRI applications". I am not sure this is correct.

Author response: We agree on this and has changed this paragraph to:

“However, while conventional MRI is sensitive to detect focal lesions in brain tissue, it cannot evaluate non-lesional changes as NAWM pathology (7). For this purpose, complementary MRI applications are required.”

7) Based on the results, is DMF improving metabolism since no change in metabolites conc. were observed in the longitudinal scan? What am I missing here?

Author response: Since we did not have a longitudinally followed control group with MS patients, we cannot argue that our findings of unchanged metabolites during the three-year follow-up is because of a stabilizing effect of DMF. Further, for the same reason, we cannot say in what way DMF affects the metabolism in NAWM of MS patients. Instead, we elaborate on relations/patterns between different metabolites and show that these metabolite relations/patterns during the follow-up are suggestive of progressive mechanisms like gliosis in this cohort.

Reviewer #2: "Diffuse white matter pathology in multiple sclerosis during treatment with dimethyl fumarate" presents metabolite concentration estimates and ratios derived from bilateral single-voxel proton magnetic resonance spectroscopy (1H MRS) measurements in normal-appearing white matter of subgroups drawn from a cohort of 26 relapsing-remitting multiple sclerosis (MS) patients and 10 controls. Metabolites assessed include total N-acetyl aspartate, myoinositol, Glx (glutamate + glutamine), total choline, total creatine, and lactate, as well as some ratios thereof, and statistical analyses involved between-group comparisons between RRMS patients and controls at baseline, longitudinal within-patient comparisons to baseline at one-year and three-year follow-up, and calculation of correlations between individual metabolite concentrations and demographic and clinical details, as well as among metabolite concentrations. The manuscript presents some interesting data but stands to be improved in several facets of persuasiveness, rigor, and readability. My detailed comments are as follows.

Abstract
1. The use of the terms “detect” and “evaluate” together in the statement “Examination of normal-appearing white matter (NAWM) changes in MS by proton magnetic resonance spectroscopy (1H-MRS) may detect and evaluate diffuse white matter pathology that is associated with neurodegeneration” seems a bit ambiguous. “Detect” makes sense, but what is the additional utility of the word “evaluate” here?

Author response: With the term “detect” we mean the possibility to discover any changes in the NAWM suggestive of pathology, and with “evaluate” we mean the possibility to investigate changes in that pathology over time. However, we agree that the term “evaluate” is redundant and removed it.

2. Please report the effect size on the p<0.01 statistic provided for mIns differences between MS and control participants.

Author response: We have changed the sentence to:

“Patients with MS had higher concentrations of *myo*-inositol (*m*Ins) in NAWM at baseline compared with controls (mean 5.98 and 4.32, p<0.01).”

3. Why do the authors specifically mention metabolite concentration stability at one-year but not three-year follow-up here?

Author response: We changed this paragraph to:

“Metabolite concentrations during one-year (n=19) and three-year (n=11) follow-up were generally stable. The dropouts were caused by treatment switch after one year, mainly due to new MRI activity.”

4. The authors use the phrase “findings in concentrations and ratios of tNA and mIns, representing neuroaxonal integrity and astrogliosis, respectively.” This needs to be broken into more precise pieces, as the list “concentrations and ratios of tNA and mIns” contains anywhere from three ([tNA], [mIns], mIns/tNA) to five or more ([tNA], [mIns], tNA/tCr, mIns/tCr, mIns/tNA, tNA/mIns) elements, and it is not eminently clear which among these the authors intend to support the claims of neuroaxonal integrity and astrogliosis.

Author response: We have changed the Conclusion paragraph to:

“Higher concentrations of the astrogliosis marker *m*Ins in MS compared to controls, the inverse relation between MS disease duration and the neuroaxonal integrity marker tNA, as well as the consistent inverse relation between these two metabolites during follow-up, showed that non-lesional white matter pathology is present in this cohort of MS patients in early disease stages. However, metabolite concentrations during follow-up were generally stable and did not reflect differences in disease activity between patients.”

5. The author order as provided on the manuscript cover page appears not to be the same as that offered by the manuscript fulltext; perhaps this should be double-checked.

Author response: The manuscript fulltext displays the correct author order.

Introduction
1. Can the authors please provide some more precise examples and cited evidence for their claim, “However, conventional MRI is relatively insensitive to detect these alterations”? This is an important point motivating the use of 1H MRS and should therefore be expanded a bit more persuasively.

Author response: We changed this paragraph in the Introduction section to:

“White matter brain tissue that appears to be free from MS lesions using conventional MRI, that is normal-appearing white matter (NAWM), is of certain interest when investigating these pathomechanisms. This is because focal white matter lesions only partially explain the development of irreversible neurologic disability, instead diffuse NAWM pathology is shown to correlate with a progressive MS disease course (6). However, while conventional MRI is sensitive to detect focal lesions in brain tissue, it cannot evaluate non-lesional changes like NAWM pathology (7, 8). For this purpose, complementary MRI applications are required.

2. Can the authors please specify the national/regional governing body and provide a citation for the claim that dimethyl fumarate “is an oral treatment for relapsing-remitting MS (RRMS) approved in 2013.”?

Author response: We changed the sentence to:

“Dimethyl fumarate (DMF, Tecfidera ®) is an oral treatment for relapsing-remitting MS (RRMS) approved by the FDA, US, in 2013 (17).”

3. The Introduction is missing a statement of the authors’ motivating hypothesis/hypotheses.

Author response: Since this was an observational study without a longitudinally followed control group, we could not make any hypotheses about specific treatment effects of DMF. However, we have changed the last paragraph in the Introduction to the following for clarification:

“Here we present an observational, up to 3-year long follow-up study, on periventricular NAWM 1H-MRS metabolite concentrations in patients with MS treated with DMF. The aim was to evaluate metabolites associated with diffuse white matter pathology and disease progression, but also to investigate if different clinical disease courses were reflected in metabolite changes during follow-up. Our hypothesis was that pathologic NAWM metabolite changes is detectable despite the absence of a progressive disease course.”

Methods
1. Many important and potentially interesting aspects of spectral data acquisition, processing, and data quality seem to be missing in this report. Overall, it is very strongly recommended that the authors include in their supplemental information a table reporting the details suggested for inclusion in papers involving magnetic resonance spectroscopy, per the recent expert consensus recommendations on minimum reporting standards in MRS: <https://pubmed.ncbi.nlm.nih.gov/33559967/>

Author response: Thanks for pointing this out. We have added information regarding the method according to the recommendations in the consensus paper.

2. Can the authors please specify more explicitly in the Methods text exactly how “disease duration” was defined in the cohort under investigation?

Author response: We have added this sentence in this paragraph:

“Disease duration was defined as time in months from first symptoms suggestive of MS to the timepoint where the baseline MRI examination was performed.”

3. The authors say that “EDSS progression was defined as an increase of EDSS… of 1.0 point from a baseline score of at least 1.0, or of 0.5 points from a baseline score of greater than 5.0.” Do they not mean “of 1.0 point from a baseline score greater than 1.0 but less than or equal to 5.0” here?

Author response: We do mean this, the higher the baseline EDSS score, the lower the progress in EDSS points to fulfill the definition of EDSS progression.

4. It is recommended that the unconventional nomenclature “tGlx” be replaced with “Glx” as per the usual name for glutamate + glutamine.

Author response: We have changed “tGlx“ to “Glx” consistently throughout the manuscript and in tables/figures.

5. Unless the profusion of correlations tested in this paper was all driven by evidence-based hypothesis explicated in the Introduction, which does not seem to be the case, it is strongly recommended that the statistics reported be filtered by correction for multiple comparisons.

Author response: We do acknowledge the risk for randomly significant correlations when performing multiple calculations. However, the correlations we highlight were not randomly scattered but showed a consistent pattern (several with a p-value of < 0.01) which we argue cannot be caused by chance. We present all p-values so that the readers themselves can make their own judgement of the significance level.

To clarify, we added this sentence at the end of the Statistics section: “Correction for multiple comparisons was not done.”

6. It is recommended that the authors spell out the shorter MR sequence designations (e.g. TSE, TFE, T2w) upon first use.

Author response: Thanks for pointing this out we agree and have changed the text accordingly.

7. It is also recommended that the authors maintain formatting consistency in their discussion of sequence timings. For example, “inversion time” and “echo time” are spelled out without provision of the usual abbreviations TI and TE, while “total acquisition time” is associated with abbreviation TA. For completeness and readability it is recommended that the authors also follow here the same convention as for sequence names, i.e. spelling out and providing an abbreviation upon first use and then using just the abbreviation thereafter.

Author response: Thanks for pointing this out. The method section has been edited.

8. Were 1H-MRS voxels isotropic? Please specify or provide dimensions (ideally in mm or cm) along each axis.

Author response: Yes, the voxel size was 13*13*13mm^3^, this has been added to the text.

9. The referenced absolute quantification method is appreciated for its detail, but some questions regarding spectral preprocessing and analysis remain. Were spectra eddy-current-corrected? If so, how? Were individual traces frequency- and phase-aligned? How many complex points did they contain, and was any line broadening or other apodization or zero-filling employed? What metabolites were included in the fit basis sets and why? Were default LCModel fit parameters including simulated macromolecules and lipids employed, or were any adjustments made? Together with my note about minimum reporting standards in point 1, including these details would be helpful in evaluating the comparability of the 1H-MRS methods used to current and future published work.

Author response: Except for setting the ATTH2O parameter as described in (Tisell et al MRM 2013) and setting “Do eddy correction” to NO since eddy current correction was already performed on the MR system using the water reference the default parameters of LCModel was used.

10. How were basis sets produced, and are the authors confident that they accurately represent the pulse sequence at hand? It appears from the cited reference (21) that they were derived from experimental measurements of phantoms, but a brief note in the methods section to this end would be of merit.

Author response: We used a simulated basis set provided by Stephen Provencher.

11. Were T2w hyperintensities in the 1H-MRS voxels also assessed in the control group? If so, why not?

Author response: T2w hyperintensities were also assessed in the control group and only voxels that were free from hyperintensities were included in the analyses.

12. What do the authors mean by “synthetic T2wMRI volume”? From which image(s) was lesion segmentation performed? This should be specified more clearly, and ideally a figure demonstrating a representative image and manually segmented lesion volume would also be provided in either the main text or supplement.

Author response: On all patients a qMRI volume using the QRAPMASTER method was measured the qMRI maps of R1, R2 and PD which are used to scale the LCModel calculated concentrations to absolute concentrations. Moreover, these methods allow generation of synthetic weighted MR images that are inherently co-registered with the R1, R2 and PD volume. And since the MRS VOI already are co-registered to the qMRI maps we used the synthetic T2w images from the QRAPMASTER measurement.

13. Why did the authors use a seemingly predetermined mix of parametric and non-parametric statistical models? Can they please justify this decision, explaining in particular why it was not instead made case-by-case according to the results of normality testing for each set of variables to be modeled?

Author response: Based on our previous work with MRS-metabolite concentrations as well as the distribution of the current data set, we know that these metabolites are normally distributed and therefore parametric statistical tests were used when analyzing these metabolites pairwise and associations between them. On the contrary, variables like CSF markers, age and disease duration were not normally distributed and therefore non-parametric statistical tests were used when analyzing these variables or when analyzing mixed data as for example correlations between MRS metabolites and disease duration.

14. Can the authors please provide a reference for the independent samples median test? Why did they choose this model for inferential statistical tests on age and disease duration in retained versus dropout patients as opposed to either Mann-Whitney U test or independent samples t-test as for the other variables?

Author response: Thanks for pointing this out. We should instead have used the Mann-Whitney test here too (data on age and disease duration was not normally distributed, hence this non-parametric test). We have now done this and revised the text to the following:

Statistics section: “Differences in age and disease duration between patients with a complete 3-year follow-up and dropout patients, were investigated using the Mann-Whitney U test.”

Results section: “Comparing baseline characteristics between dropout patients and patients with a complete 3-year follow-up on DMF treatment, showed that dropout patients were younger (median 30 years old, n=8 versus 44 years old, n=18, p=0.04), but the disease duration time was not significant different (median 22 months, n=8 versus 71 months, n=17, p=0.09).”

Discussion section: “The dropout rate however introduces a possible selection bias in the 3-year follow-up results, since dropout patients in general were younger compared with patients that completed the 3-year long follow-up.”

15. Similarly, why were metabolite concentrations vs. age or disease duration correlations assessed nonparametrically while inter-metabolite concentration correlations were assessed parametrically? Can the authors justify these decisions with normality tests?

Author response: Please see author response to question 13.

Results
1. It is not clear what level of spectral data quality (as measured by full width at half maximum FWHM and/or signal to noise ratio SNR on singlets like creatine or NAA, as well as Cramer-Rao Lower Bound CRLB on metabolite fit estimates) was achieved in these results and whether this variable may have differed in the different groups and time points under study.

Author response: CRLB was not used since this was a group study. FWHM was assessed on the NAA resonance.

2. Notably, did spectral data quality (particularly singlet FWHM or SNR) itself correlate with disease duration and/or age?

Author response: This was nothing we intended to analyze thus we did not save the FWHM and SNR estimation with the patient data. However, we could retrieve FWHM, SNR and an approximate age (calculated from the patient's birth year) from the saved LCModel table file.

This was used to calculate spearman correlations. Spearman r for age vs SNR was –0.36 and r for age vs FWHM was 0.28 (see figure above). However, how this effect the analysis is difficult to assess. Since the patients are their own controls in the paired analysis age effect are controlled for. And, looking at the scatter plots age vs SNR and FWHM show that even if there is a correlation there is not very much of the variation of the SNR and FWHM that are explained by the relation age vs SNR and FWHM.

3. Were inter-metabolite correlations also examined in controls, and are the authors confident that those found in patient groups were indeed abnormal by comparison rather than simply reflecting banalities of voxel composition (e.g., tNA and mIns concentrations are inversely correlated because they might simply reflect grey-white matter composition in all participants, even controls)?

Author response: This is a highly relevant question. We have now performed inter-metabolite concentration calculations also in controls, but then only found an inverse relation between mIns and Glx. The consistent inverse correlation between mIns and tNA found in patients was in other words not present in the controls, indicating that this pattern represents NAWM pathology.

We have added this sentence to the Results section: “When performing the same inter-metabolite correlation analyses in controls the only association found was an inverse relation between mIns and Glx (r= -0.69, p=0.028).”

4. The authors claim, “…we also calculated ratios between concentrations of tNA and tCr (=tNA/tCr), mIns and tNA (=mIns/tnA), tCho and tNA (=tCho/tNA), as well as tGlx and tNA (=tGlx/tNA), since these ratios have been suggested of prognostic value (22, 23, 24).” This is a rather large claim to unpack, and it is not clear how each of the cited works supports its application to the multiple concentration ratios presented here. It is therefore recommended that this discussion be moved to the Introduction, with more targeted evidence-based persuasion of the readers that these examined concentration ratios are actually of biological or clinical value.

Author response: We agree on this comment and added the following section in the Introduction to explain the rational (including references) for calculating these ratios:

“In addition to evaluating metabolites separately, ratios of metabolite concentrations have also been shown to reflect pathological processes in cerebral tissues. In a 4-year long 1H-MRS study of mechanisms of progressive MS it was found that the ratio between mIns and tNA in NAWM had consistent predictive power on brain atrophy and neurological disability evolution (13), and in a 5-year long follow-up study of different subtypes of MS, an increase in the ratio of glutamate and tNA in NAWM was shown to be associated with a decline in brain volume (14). Additionally, in a meta-analytic review the ratio between tNA and glial cell marker creatine was suggested to be a surrogate measure of neuroaxonal integrity (15), and the ratio between the glial cell marker choline and tNA has been found a putative marker of progressive MS onset (16).”

5. The authors state, “There were no differences in metabolite concentrations at the one-year follow-up comparing patients with NEDA or EDA during the same time period (data not shown).” Can the authors please provide a table supporting this result in the supplemental information?

Author response: We have added a supplemental table (S3) with data on metabolite concentrations and ratios with respect to NEDA and EDA during the 1- and 3-year follow-up respectively.

We added this sentence to the Statistics section: “The independent samples t-test was used for comparing differences in metabolite concentrations and ratios of metabolite concentrations in relation to disease activity during follow-up.”

We added this sentence to the Results section: “There were no differences in metabolite concentrations or metabolite concentration ratios at the 1-year or 3-years follow-up, comparing patients with NEDA or EDA during the corresponding time period (S3 Table).

6. Did the authors assess metabolite concentration ratio (e.g., tNA/tCr, mIns/tNA, tCho/tNA, tGlx/tNA) differences between NEDA and EDA patients at one-year or three-year follow-up?

Author response: Please see answer to question 5 above.

Discussion
1. The authors claim, “Glial cells as oligodendrocytes, astrocytes and microglia have shown to be highly involved in the regulation and production of extracellular glutamate levels (34, 35, 36).” Can they please move each of these citations into the parts of this general claim that they are supporting? It is not clear, in other words, which reference is providing evidence of involvement in extracellular glutamate handling for which of the glial cell types.

Author response: We have changed this sentence to: “Glial cells such as oligodendrocytes (36), astrocytes (37) and microglia (38) have been shown to be highly involved in the regulation and production of extracellular glutamate levels.

Figures

1. Optimally the authors would display an overview, as by superposition or averaging of datasets, of all the spectra collected in this mixed cross-sectional and longitudinal study. At the very least, they should include a figure demonstrating voxel placement; a sample spectrum, ideally from each group; and the fit methods employed (i.e. one dataset with overlaid model and fitted basis components, including calculated baseline, any macromolecule or lipid models, and residual).

Author response: Figure 1 has been added showing typical spectrum at each time point.

2. It is stated, “This notion is also supported by a longitudinal case-control MRS study showing that the ratio of mIns and tNAA in NAWM had consistent predictive power on brain atrophy and neurological disability evolution (23).” Can the authors please specify the study cohort in which this was shown to be the case in the cited work so that readers can better evaluate its relationship to the population investigated in the present paper?

Author response: We have changed this part in the Discussion to the following:

“This notion is also supported by the findings from a longitudinal case-control study investigating if changes in ^1^H-MRS metabolites in NAWM and gray matter could predict brain volume loss and disability progression (23). In this study, 59 MS patients with a mean age of 42 years, a mean disease duration of 10 years and a median EDSS score of 1.5, constituted a discovery cohort that was followed for a mean time of 3.5 years. The ratio of mIns and N-acetylaspartate in NAWM had consistent predictive power on brain atrophy and neurological disability evolution, and the same result was also found in the confirmatory cohort of 220 MS patients with a mean follow-up of 3.6 years.”

Supplementary Information
1. Similarly to my note on the Methods---PLOS ONE has a broad readership, and it is therefore especially recommended that acronyms and abbreviations employed in Supplemental File 1. Clinical protocol be spelled out explicitly either upon first use or in a table legend.

Author response: We have changed this in the S1 Table.

Minor notes on diction and typography

While the manuscript is easily readable, it does exhibit frequent grammatical errors and would therefore benefit from a careful re-review of this aspect. Some suggested corrections include but are not limited to the following:

1. normal appearing white matter -> normal-appearing white matter
2. this observationally study -> this observational study
3. twentysix -> twenty-six
4. differences in disease activity between patients -> differences in disease activity among patients
5. The ability of these DMTS… are considerable -> The ability of these DMTs… is considerable
6. However, accumulating evidence… suggest -> However, accumulating evidence… suggests
7. enables a longitudinal in vivo detection -> enables longitudinal in vivo detection
8. our group have -> out group has
9. started at 1 October … stopped at 31 October -> started 1 October… stopped 31 October
10. A conventional MRI… were -> A conventional MRI… was
11. MRS-voxel -> MRS voxel
12. with in -> within
13. MRSexaminations -> MRS examinations
14. In this three-year long observational study -> In this three-year observational study
15. This study have limitations -> This study has limitations
16. This finding implicate that -> This finding implies that
17. no differences… were observed, which questions the -> no differences… were observed, which calls into question the
18. NEDA probably is measure far too blunt -> NEDA probably is a measure far too blunt
19. the mechanisms that underlies disease -> the mechanisms that underlie disease
20. MS patients treated with DMF findings in -> MS patients treated with DMF. Findings in

Author response: Thanks for pointing this out. We did another re-review of the text for editing grammatical errors.
