# Supplementary material for: Diffuse white matter pathology in multiple sclerosis during treatment with dimethyl fumarate—An observational study of changes in normal-appearing white matter using proton magnetic resonance spectroscopy
Source: PLoS One. 2024 Oct 21;19(10):e0309547. doi: 10.1371/journal.pone.0309547 (PMC11493296; doi:10.1371/journal.pone.0309547)
Supplement: Attachment — Submitted filename: Response to Reviewers.docx [file pone.0309547.s006.docx]

**Additional Response to Reviewers**

**Reviewer #1:**

1) Please add these info in the text
- the mean and max linewidth (R1.2)
- metabolites in basis set including simulated lipids/MM signals (R1.3)

Author response: Mean and max linewidths have been added in the end of the Methods section “MRI and post processing of ^1^H-MRS metabolite concentrations”. In addition, an explanation of used basis set has also been added to the same section.

2) The authors did not respond whether preprocessing was carried out (R1.3 and R2.9). Please also add this to text.

Author response: No extra preprocessing of the MRS data was performed, except calculation of the scaling factor ATTH2O of LCModel. The section “MRI and post processing of ^1^H-MRS metabolite concentration” section have been edited to clarify this.

3) About lactate comment (R1.5), from figure 1 and the noise level, it is hard to tell if indeed lactate is present or LCModel is fitting lipids as lactate. Was outer volume suppression pulses used in the sequence to minimize signal outside the VOI?

Author response: No outer volume suppression was used. Volume selection was only based on the slice selective RF-pulses.

4) Note that to be consistent with MRS convention; total N-acetylaspartate should be tNAA and not tNA. Please update text and figures.

Author response: On this point we do not agree with the reviewer and we are unaware if there is any consensus on MRS abbreviations. However, with tNA we mean the total of metabolite containing N-acetyl groups i.e. N-acetylaspartate + N-acetylaspartylglutamate. The second A in NAA and NAAG refers to aspartate and aspartyl, respectively, and an abbreviation of tNAA would suggest that the second A stands for either aspartate or aspartyl. Both tNAA and tNA are commonly used abbreviations in MRS studies. The manuscript, tables and figure legends have been updated according to this.

**Reviewer #2:**

The responses to my first round of revisions to this manuscript are appreciated, though some out-standing questions remain. Below are my detailed replies:

Abstract
1. The authors have adequately responded to this comment.
2. These are two group means (which are by themselves not too meaningful without associated standard deviations or errors), but what is still needed is the statistic with which this p-value is associated.

Author response: The sentence has been changed to:

“Patients with MS had higher concentrations of *myo*-inositol (*m*Ins) in NAWM at baseline compared with controls (mean 5.98 ± 1.37 (SD) and 4.32 ± 1.16 (SD), p<0.01, independent samples t-test).”

3. The authors have adequately responded to this comment.
4. The authors have adequately responded to this comment.
5. The authors have adequately responded to this comment.

Introduction
1. The authors have adequately responded to this comment.
2. The authors have adequately responded to this comment.
3. The authors have adequately responded to this comment, though “changes is” should be “changes are.”

Author response: This grammatical error has been edited.

Methods
1. Ideally the relevant methods would be fully tabulated and provided in complete detail according to MRSinMRS format, especially because this greatly facilitates inclusion of the present study in methodological meta-analyses. The current version is, however, an improvement over the first. “Preformed” should be “performed.”

Author response: This grammatical error has been edited.

2. The authors have adequately responded to this comment.
3. It should be specified with more precision in the manuscript that the authors do mean “of 1.0 point from a baseline score greater than 1 and less than 5” if this is indeed the case, as right now the language is a bit misleading.

Author response: We agree that this sentence is misleading and changed to:

“EDSS progression was defined as an increase in EDSS of 1.5 points from a baseline score of 0, of 1.0 points from a baseline score of at least 1.0, or of 0.5 points from a baseline score of 5.0 or higher.”

4. The authors have adequately responded to this comment.
5. The authors have adequately responded to this comment.
6. The authors have adequately responded to this comment.
7. The authors have adequately responded to this comment.
8. The authors have adequately responded to this comment.

9. I still recommend, in line also with point 1 here in my Methods comments, increased detail provided for the manuscript’s descriptions of spectral preprocessing and analysis methods. If averages were aligned and averaged on the scanner instead of exported and processed afterward, this should be specified. If they were not aligned for phase or frequency drift and simply averaged in a homebrew MATLAB script then that should be specified. Right now it is not clear how “transients were averaged” as written in the text, as many options for this exist in the realms of alignment and weighting and it is not clear still what was done. Details such as eddy-current correction as provided in the rebuttal should also be in the text.

Author response: The measurements were performed on a Philips Ingenia MR system using the provided PRESS sequence. Raw data was saved and exported using the PAR/REC export tool in .SPAR/ .SDAT format. This data is already averaged and eddy current corrected. The section “MRI and post processing of ^1^H-MRS metabolite concentrations” section have been edited to clarify this.

10. Similarly, it should be explicitly noted in the text that the basis set provided was a boilerplate file by Stephen Provencher and not, presumably, tailored to the pulse shapes and exact timings of the sequence actually employed for the acquired data fitted, as this discrepancy could have bearing on the results reported.

Author response: Information about the basis set has been added in the method section “MRI and post processing of ^1^H-MRS metabolite concentrations “.

11. It should be specified more clearly in the text that control group voxels were also assessed for T2w hyperintensities and excluded if such were found.

Author response: This have been clarified in the manuscript: “In the first step all MRS voxels placements both in patients and controls were visually inspected and measurements that were in close proximity of a lesion were selected for step two.”

12. It should be specified more clearly in the text and not just the author rebuttal what the authors mean by synthetic T2wMRI volume and how its various output components were used as this is an interesting detail that is not immediately clear to those not familiar with QRAPMASTER.

Author response: It has been added that the syntethic T2 volumes were generated from the qMRI data.

13. Can the authors please explicitly mention in the text whether and how this normality testing was done to justify the decision to test between-group differences with parametric or nonparametric approaches?

Author response: We added this sentence at the top in the Statistics section:

“Data distributions for variables were tested for normality with Kolmogorov-Smirnovs test and the result then guided the following use of either parametric or non-parametric tests.”

14. The authors have adequately responded to this comment, though, again, the authors should not report a p-value without the associated test statistic.

Author response: We changed this paragraph to:

“Comparing baseline characteristics between dropout patients and patients with a complete 3-year follow-up on DMF treatment, showed that dropout patients were younger (median 30 years old, n=8 versus 44 years old, n=18, p=0.04, Mann-Whitney U-test), but the disease duration time was not significant different (median 22 months, n=8 versus 71 months, n=17, p=0.09, Mann-Whitney U-test). Dropout patients also had lower concentrations at baseline of tCr and tCho (mean difference 0.42, p=0.008 and 0.37, p=0.005, respectively, independent samples t-test) compared with patients with a complete follow-up.”

15. Thank you for this response---but similarly to 13: Can the authors please explicitly mention in the text whether and how this normality testing was done to justify the decision to test between-group differences with parametric or nonparametric approaches?

Author response: Please see answer to 13 above.

Results
1. Was NAA FWHM consistent across groups and time points? While not necessary to report, it would strengthen the authors’ data, though see point 2.

Author response: Unfortunately, the FWHM was not a parameter exported to the statistic file where information of age and group were stored. Thus, only an evaluation of the entire groups FWHM was performed. However, considering the limited value of LCModels discretized value of FWHM, we do not see the point of evaluating this further.

2. Provencher’s LCModel discretizes its singlet FWHM measurements rather roughly, making a correlational analysis using this metric difficult. The authors have adequately responded to this comment, though in the future it would be ideal if they had a pipeline to measure singlet SNR FWHM outside of LCModel so as not to lose potentially informative precision.

Author response: Yes, we agree that other tools preferably working over several vendors would be interesting, so that a more precise and informative method for calculating FWHM and SNR could improve the analysis of MRS data.

3. The authors have adequately responded to this comment.
4. The authors have quite adequately responded to this comment.
5. The authors have adequately responded to this comment.
6. The authors have adequately responded to this comment.

Discussion
1. The authors have adequately responded to this comment.

Figures
1. The authors have adequately responded to this comment.
2. The level of detail provided now is perhaps a bit much (I should have clarified that what I was after in my original comment was MS clinical course), but the response is appreciated.

Supplementary Information
1. The authors have adequately responded to this comment.

The authors have adequately responded to the minor notes on diction and typography.
